# Strengthening the National Reference Laboratory in the Republic of Congo: An Investment Imperative for Tuberculosis Diagnostics

**DOI:** 10.3390/tropicalmed11010023

**Published:** 2026-01-13

**Authors:** Darrel Ornelle Elion Assiana, Franck Hardain Okemba-Okombi, Salomon Tchuandom Bonsi, Freisnel Hermeland Mouzinga, Juliet E. Bryant, Jean Akiana, Tanou Joseph Kalivogui, Alain Disu Kamalandua, Nuccia Saleri, Lionel Caruana, Hugues Traoré Asken, Dissou Affolabi

**Affiliations:** 1 Faculté des Sciences et Techniques, Université Marien Ngouabi, Brazzaville P.O. Box 69, Congo; freisnelm@gmail.com (F.H.M.); 23akianajean@gmail.com (J.A.); 2 Programme National de Lutte contre la Tuberculose, Brazzaville P.O. Box 1066, Congo; franckokemba@gmail.com (F.H.O.-O.); salomon.tchuandom.bonsi@undp.org (S.T.B.); alain.disu.kamalandua@undp.org (A.D.K.); 3 Laboratoire National de Référence des Mycobactéries, Brazzaville P.O. Box 1066, Congo; 4 Faculté des Sciences de la Santé, Université Marien Ngouabi, Brazzaville P.O. Box 2672, Congo; 5 Service de Pneumologie, Centre Hospitalier Universitaire de Brazzaville, Brazzaville P.O. Box 32, Congo; 6 United Nations Development Programme, Brazzaville P.O. Box 465, Congo; tanou.joseph.kalivogui@undp.org (T.J.K.); hugues.traore1@undp.org (H.T.A.); 7 Fondation Congolaise pour la Recherche Médicale, Brazzaville P.O. Box 2672, Congo; 8 The Global Fund to Fight AIDS, Tuberculosis and Malaria, P.O. Box 1218 Geneva, Switzerland; juliet.bryant2@theglobalfund.org (J.E.B.); nuccia.saleri@theglobalfund.org (N.S.); lionel.caruana@theglobalfund.org (L.C.); 9 Direction des Technologies de la Santé, Ministère de la Santé et de la Population, Brazzaville P.O. Box 1066, Congo; 10 National Teaching Hospital for Tuberculosis and Pulmonary Diseases, Cotonou P.O. Box 817, Benin; affolabi_dissou@yahoo.fr; 11 Faculty of Health Sciences, University of Abomey-Calavi, Cotonou P.O. Box 526, Benin; 12 Supranational Reference Laboratory in Cotonou, Cotonou P.O. Box 817, Benin; 13 National Tuberculosis Program, Cotonou P.O. Box 321, Benin

**Keywords:** Tuberculosis, drug-resistant TB, National Tuberculosis Reference Laboratories, surveillance, Republic of Congo

## Abstract

National Tuberculosis Reference Laboratories (NTRLs) are central to tuberculosis (TB) control programs. Between 2018 and 2024, the Republic of Congo, a country of 6 million inhabitants, achieved a transformative strengthening of its TB diagnostic system, coordinated by the NTRL. Strategic investments, supported mainly by international partners, enabled a substantial decentralization of services, expanding the diagnostic network from 38 to 113 diagnostic and testing centers and increasing GeneXpert sites from 3 to 31. The expansion of the diagnostic network and specimen referral system was associated with a reduced structural gap in diagnostic coverage by extending access to GeneXpert testing to a larger number of peripheral and previously underserved centers. Critically, the establishment of a BSL-3 laboratory and the deployment of advanced assays like Xpert MTB/XDR ended the reliance on overseas testing by introducing in-country capacity for multidrug-resistant and pre-extensively drug-resistant TB detection. These systemic improvements were associated with significant positive outcomes, including an annual molecular testing surging from 11,609 in 2022 to over 27,000 in 2024 and bacteriological confirmation rates rising from 34 to 73%. This comprehensive laboratory systems strengthening, which also facilitated cross-programmatic initiatives like HIV and Mpox testing integration, underscores how sustained investment in infrastructure, logistics, and quality management is fundamental to improving case detection, surveillance, and progress toward the WHO End TB Strategy milestones.

## 1. Introduction

Tuberculosis (TB) remains the leading cause of death from a single infectious agent. In 2024, the World Health Organization (WHO) estimated that 10.8 million people developed TB and 1.25 million died from the disease, including 161,000 deaths among people living with HIV. Multidrug-resistant TB (MDR-TB) further complicates this picture, posing a critical threat to global health security and antimicrobial resistance (AMR) control. Despite available tools, progress towards the End TB strategy targets is stalling, underscoring the WHO’s emphasis on robust laboratory networks for TB detection, including MDR/TB, treatment monitoring, and policy guidance [1].

National Tuberculosis Reference Laboratories (NTRLs) form the cornerstone of the national TB response by serving as the highest authority for testing algorithms and quality assurance. Their mandate also includes performing specialized diagnostics like drug susceptibility testing, conducting research, evaluating new tools, and overseeing laboratory workforce development.

Without well-resourced National Tuberculosis Reference Laboratories, surveillance systems for TB cannot provide the reliable data required for effective national planning or meaningful contributions to global monitoring [2]. The value of such laboratories is demonstrated by the WHO’s supranational reference laboratory (SRL) network for TB, which strengthens surveillance through proficiency testing, method harmonization, and rapid drug resistance detection. In Europe, NTRLs are essential for providing timely, standardized data on drug resistance, which directly guides the public health response [3,4]. NTRLs supervise external quality assessment (EQA) and facilitate the ongoing evolution of national TB diagnostic algorithms. Over the years, they have played a major role in promoting the adoption of novel technologies, such as Line Probe Assays (LPAs), molecular WHO-recommended diagnostics (GeneXpert, TrueNat, etc.), and targeted genomic sequencing. NTRLs act as both policy anchors and technical leaders. By ensuring diagnostic accuracy within national reporting systems, NTRLs support the larger surveillance system in monitoring changes in TB epidemiology and better understanding the efficacy and impact of disease control interventions [2,3,4].

These functions are particularly critical in sub-Saharan Africa, where health systems contend with challenges such as underfunding, limited laboratory infrastructure, and a historical reliance on external diagnostic and testing centers for advanced testing. The absence of local capacity for *Mycobacterium tuberculosis* complex culture drug susceptibility testing (DST) and advanced technologies such as sequencing platforms has historically delayed diagnosis and compromised patient management [5,6].

The Republic of Congo remains a high TB burden setting, with an estimated incidence of 302 cases per 100,000 inhabitants in 2024. Prior to 2018, TB diagnosis was conducted across 38 diagnostic and treatment centers nationwide, largely relying on smear microscopy as the primary diagnostic method. Approximately 90% of bacteriologically confirmed TB cases were diagnosed by microscopy alone. Molecular testing capacity was limited, with no fully functional National Reference Laboratory and no standardized national algorithm for drug-resistant TB diagnosis. TB nucleic acid amplification tests were either unavailable or used only in a limited manner for selected high-risk patients (available in only three centers across a country of around 6 million inhabitants), constraining early detection of drug-resistant TB.

Targeted investments in NTRLs have yielded measurable gains in TB disease control across diverse settings. In India, scaling up rapid molecular diagnostics at reference laboratories significantly improved the detection of rifampicin resistance, directly informing national treatment policies [7]. Similarly, a WHO-supported collaboration with a supranational reference laboratory in Uganda strengthened proficiency testing, leading to substantial improvement in DST reliability [8]. In Portugal, adoption of whole-genome sequencing (WGS) at the NTRL shortened resistance profiling turnaround times and enhanced outbreak investigations [9]. In Benin, the SRL in Cotonou has reinforced quality assurance across national and regional TB networks, supporting EQA and acting as a training hub for Central and West Africa [10]. Collectively, these examples demonstrate that strategic investments in laboratory systems strengthening, NTRLs, and SRLs lead to meaningful improvements in TB disease control and patient outcomes.

The primary objective of this study was to undertake operational research and program evaluation to assess advancements in TB diagnostic capacity and the performance of the laboratory network in the Republic of Congo between 2018 and 2024. As key indicators of progress, we specifically focused on diagnostic coverage, drug-resistant TB detection, specimen referral, HIV integration, quality assurance, and regional research collaboration.

## 2. Methods and Materials

### 2.1. Study Design

This study was conducted as an operational research and program evaluation using a retrospective observational design. Routinely collected, aggregated programmatic data from the national TB program were analyzed to describe trends in TB diagnostic capacity, laboratory network performance, and specimen referral systems between 2018 and 2024.

### 2.2. Setting

This study was conducted in the Republic of Congo (which has a population of approximately 6.1 million). The intervention was implemented on a national scale, encompassing the full network of public health diagnostic and testing centers that offer TB diagnostics.

### 2.3. Data Source

All data analyzed in this study originated from routinely collected programmatic sources of the National TB Program and the DHIS2 surveillance system. Diagnostic and treatment centers collect patient-level data, which are aggregated quarterly at the departmental level and submitted to the national TB program. We particularly considered the following:

✓ Annual TB case notification reports (bacteriologically confirmed, drug-resistant).
✓ Laboratory service data, such as test volumes.
✓ Records of the diagnostic network, including the number and distribution of Diagnostic and Treatment Centers (DTCs).

Number of DTCs involved in referral TB-sample data quality assurance is performed at multiple levels, including verification at the diagnostic center level, consistency checks during departmental aggregation, and national-level review prior to analysis. Missing or inconsistent data are flagged and verified with reporting sites when feasible. Analyses were conducted using available aggregated data; the proportion of missing information was low and did not materially affect interpretation of temporal trends.

Documentation on project implementation, procurement, and technical assistance is provided with the Global Fund and other partners (WHO, UNDP, Red Cross).

### 2.4. Quality Management System Records

Data from the External Quality Assessment (EQA) programs and the WHO SLIPTA audit report were also used to measure improvements in laboratory quality.

### 2.5. Laboratory Strengthening Intervention and Definitions of Key Outcomes of Interest

With technical and financial support from the Global Fund, WHO, UNDP, and the Red Cross, the NTRL was established and operationalized to enhance the national diagnostic capabilities. Key interventions included the following:

✓ Infrastructure development with construction, equipping, and biosafety certification of central NTRLs, including BSL-2 and BSL-3 laboratories, to enable safe processing of DR and TB specimens and culture.
✓ Diagnostic network decentralization with a strategic expansion of TB diagnostic network at the countrywide level.
✓ Scale-up of molecular WHO-approved rapid diagnostic (mWRD) platforms.
✓ Implementation of a national specimen transport system ensuring timely sample referral and result feedback.
✓ Training and capacity building for laboratory staff in biosafety, molecular diagnostics, and data management.
✓ Establishment of a national quality management system, including external quality assessment (EQA) and WHO SLIPTA audits.

Key outcomes of interest included the following:

✓ Bacteriological confirmation rate, defined as the proportion of notified TB cases with laboratory confirmation;
✓ Diagnostic coverage site and geographic distribution, defined as the number of diagnostic and testing sites able to perform TB diagnosis across the country;
✓ Drug-resistant TB detection, measured by the annual number and proportion of bacteriologically confirmed TB cases with DR detected by molecular assays;
✓ Specimen referral performance, assessed by the number of samples referred through the national referral network and the expansion of referral linkages over time;
✓ HIV–TB service integration, measured by the proportion of TB patients with documented HIV testing;
✓ Laboratory quality assurance, evaluated based on its existence and then by the number of sites participating in that EQA;
✓ Regional research and laboratory collaboration, measured by the establishment and utilization of national and regional laboratory partnerships.

All indicators were calculated using denominators as defined in routine national reporting.

### 2.6. Data Analysis

All data were entered into Microsoft Excel 365 and analyzed using GraphPad Prism 8 and the Joinpoint Regression Program (v5.2.0, National Cancer Institute, Bethesda, MD, USA) [11]. Descriptive statistics summarized key programmatic indicators, including the number of diagnostic centers and GeneXpert sites, Xpert MTB/RIF and MTB/XDR testing volumes, and HIV testing coverage among TB patients. Joinpoint regression analysis was applied to evaluate temporal trends, identify statistically significant changes in linear trends, and calculate annual percentage change (APC) with 95% confidence intervals (CIs), with *p*-values < 0.05 considered significant. This was based on a grid search method with a minimum number of two observations from a Joinpoint. The parametric method and permutation test were employed for model selection. In this model, period in calendar years was the only independent variable, while all the other variables were treated as dependent, assuming homoscedasticity over time for each test. The calculated APC represents the average yearly rate of change, reflecting the trend’s slope over time [12].

Additional analyses included calculation of absolute and relative changes in TB case detection, MDR/Pre-XDR TB diagnoses, and referral network expansion.

### 2.7. Ethical Considerations

As this study was based on the analysis of aggregated, routine program-level data for public health monitoring and evaluation, formal individual patient consent was not required. All data were handled in strict accordance with national health data protection policies, ensuring patient confidentiality was maintained. All activities described were conducted under the authority and oversight of the Republic of Congo’s National Tuberculosis Program. For any questions regarding research integrity, please contact Prof. Franck Hardain OKEMBA-OKOMBI, Director of the National Tuberculosis Control Program, franckokemba@gmail.com.

## 3. Results

### 3.1. Expansion of the National Tuberculosis Diagnostic NETWORK

Between 2018 and 2024, the Republic of Congo achieved substantial progress in strengthening its TB diagnostic network under the coordination of the NTRL. Before the NTRL was established and began operations in 2018, there were only 38 to 40 diagnostic and testing centers across the country.

**Figure 1:**
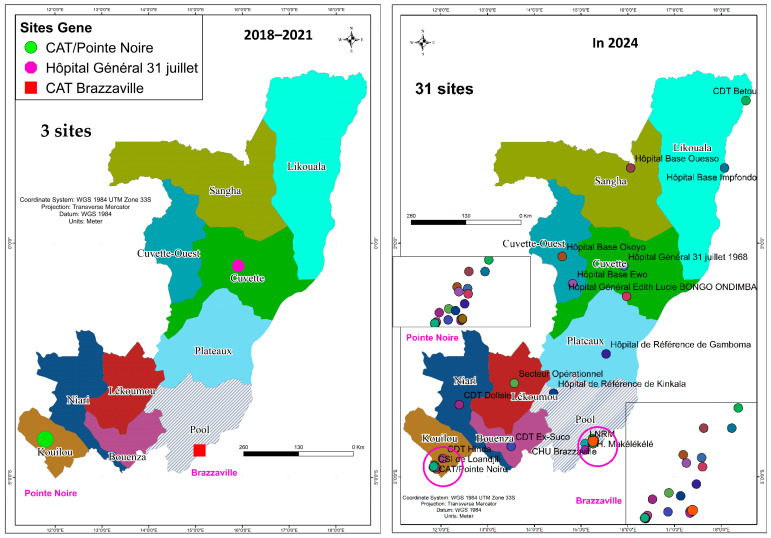
Number of GeneXpert sites in the laboratory network between 2018 and 2024.

The NTRL now offers comprehensive TB testing services, including microscopy, GeneXpert, line probe assays (LPAs), and culture. By October 2024, the national diagnostic network had expanded to 113 diagnostic and treatment centers, all of which were equipped with smear microscopy; among these, 31 centers were additionally equipped with GeneXpert systems. This expansion was statistically significant (APC +10.9%, 95% CI: +2.5 to +20, *p* < 0.01), as shown in Table 1 and Figure 1.

The expansion in diagnostic capacity occurred alongside improvements in patient management and treatment outcomes at the national level. Treatment success significantly improved from 70 in 2018 to 87% in 2024, with a positive average annual percentage change (APC +4.4%, 95% CI: 1.9 to 7.14; *p* = 0.0076).

More notably, a significant improvement was observed in unsuccessful treatment outcomes. Between 2018 and 2024 the proportion of patients lost to follow-up declined substantially, with an APC of −12.7% (95% CI: −19.1 to −5.1; *p* < 0.05).

### 3.2. Establishing In-Country Capacity for MDR/Pre-XDR TB Testing

Until 2021, only three [3] diagnostic and testing centers nationwide offered mWRD. By 2024, molecular testing capacity expanded to 31 diagnostic centers performing mWRD across 11 of 12 departments, decentralizing testing. The proportion of bacteriologically confirmed cases rose to 70% (APC +6.8%, 95% CI 2.5–11.4, *p* = 0.006; see Table 1).

A major step toward drug-resistant TB control was achieved in 2022, when the NTRL acquired a ten-color GeneXpert system enabling in-country detection of multidrug-resistant (MDR) and pre-extensively drug-resistant TB.

Between 2022 and 2024, the annual number of Xpert MTB/RIF or Ultra tests increased from 11,609 in 2022 to 27,318 in 2024, with corresponding increases in TB-positive detections from 4185 to 7179 (representing an annual percentage change (APC) of 53.46%). Rifampicin-resistant TB detections rose from 215 cases in 2022 to 273 cases in 2024, as shown in Table 2.

Second-line drug resistance testing using the Xpert MTB/XDR assay was performed among rifampicin-resistant TB cases from 2023 onward. In 2023, Xpert MTB/XDR testing was performed in 102/250 RR patients and identified 17 mono-resistant, 49 multidrug-resistant (MDR), and 38 pre-XDR cases. In 2024, these numbers increased to 67 mono-resistant, 90 MDR, and 88 pre-XDR cases in 243/273 RR patients. See Table 2.

### 3.3. Expansion of the Sample Transport and Referral Network

To enhance access to diagnostics across the country, a national specimen referral network was established, operating on a hub-and-spoke model with defined circuits, fixed schedules, and trained drivers to ensure safe and temperature-controlled transport. This system enables the referral of respiratory specimens (sputum) from peripheral sites without GeneXpert to facilities equipped with GeneXpert for TB testing, facilitating timely diagnosis and improving linkage to care.

In 2022 (year of establishment), 123 sputum samples were referred from 10 diagnostic and treatment centers (DTCs) in 2 departments. The number rose to 2154 samples from 29 DTCs in 8 departments in 2023, and 5160 samples from 49 DTCs in 11 departments in 2024 (Table 3).

This system, initially established for TB diagnosis, has also proven adaptable for other programs, including emergency monkeypox sample transfers in 2024 (during a pandemic) and potential HIV viral load and early infant diagnosis specimen transport.

### 3.4. Integration of HIV Testing in TB Services

Between 2018 and 2024, the proportion of TB patients tested for HIV increased from 29% to 90%, with an annual percentage change (APC) of 26.2 (95% CI: 2.6–52.6; *p* < 0.05) Table 1. This proportion remained consistent at approximately 26% for several years prior to 2018.

### 3.5. Implementation of External Quality Assessment (EQA) Systems

By late 2024, a comprehensive External Quality Assessment (EQA) framework was established under NTRL supervision for all TB testing centers nationwide:

- The Supranational Reference Laboratory (SRL) in Cotonou (Benin) provides EQA for microscopy to the NTRL.
- Longhorn Vaccines and Diagnostics, LLC (USA), oversees EQA for GeneXpert testing to the NTRL.
- The NTRL, in turn, provides EQA for both microscopy and GeneXpert to all active TB centers across the Republic of Congo.

Prior to the establishment of the NTRL, external quality assurance (EQA) for microscopy was limited to a single in-country diagnostic and treatment center (DTC) by the Supranational Reference Laboratory (SRL) in Cotonou, with no subsequent cascade of EQA to other diagnostic and testing centers. All participating diagnostic and treatment centers were enrolled in a nationally coordinated EQA scheme. During the initial phase, laboratories received raw EQA panels for baseline assessment prior to formal training. These baseline assessments informed targeted, NTRL-led training and corrective actions across participating sites. As this phase was designed for capacity assessment and quality improvement, rather than standardized performance evaluation, validated laboratory performance indicators were not generated during the study period.

### 3.6. Research Collaborations

Strategic partnerships have expanded substantially. The NTRL currently trains an increasing number of technicians and biologists’ students and collaborates with the following:

- National universities: Marien Ngouabi University and Denis Sassou Nguesso University.
- Public research institutes: National Public Health Laboratory, University Hospital of Brazzaville, National Institute for Health Research, and Institute for Research in Exact and Natural Sciences.
- Private institutions: Congolese Foundation for Medical Research.

Regionally, the NTRL contributes to ongoing multinational studies with the National Reference Laboratory for Mycobacteria (DRC), CERMEL (Gabon), the Pasteur Institute of Cameroon, and IRESSEF (Senegal). These collaborations position NTRL as an emerging research hub for Central Africa.

### 3.7. Achievements

Major overall achievements are summarized in Table 1, Table 2 and Table 3. By 2024, the NTLR ensured national coordination of TB diagnostics and drug resistance surveillance, culture-based therapeutic monitoring, quality assurance across the laboratory network, and integration of advanced molecular epidemiology. The NTLR also enabled systematic HIV diagnosis in TB patients, leading to a significant increase in HIV testing rates among this group over time.

## 4. Discussion

The primary objective of this study was to evaluate the expansion and strengthening of the National Tuberculosis Reference Laboratory network in the Republic of Congo between 2018 and 2024, focusing on diagnostic coverage, drug-resistant TB detection, specimen referral, HIV integration, quality assurance, and regional research collaboration. This evaluation provides critical insight into the progress achieved, identifies persisting gaps, and informs strategies to accelerate progress toward the WHO End TB targets.

The modernization of the National Tuberculosis Reference Laboratory in the Republic of the Congo has markedly reshaped the country’s TB diagnostic landscape. In less than a decade, the country leapfrogged from a limited diagnostic capacity, reliant on just three GeneXpert sites and outsourcing drug resistance management, to a nationally coordinated network equipped for comprehensive bacteriological testing. These achievements underscore how strategic investments in laboratory systems, human resources, and sample transport are associated with increased case detection, improved surveillance, and faster patient management [13]. The expansion of NTRL-led diagnostic capacity and the observed improvements in key treatment indicators are consistent with strengthened diagnostic capacity at the programmatic level. The positive, non-significant trend in treatment success, coupled with the significant reduction in loss to follow-up, suggests an alignment between improved diagnostics capacity and a more effective care cascade. This highlights how investing in laboratory networks improves diagnostics and is temporally associated with better patient management and outcomes, which is vital for controlling TB. The rapid decentralization of services, as demonstrated by the increase in diagnostic network facilities from 38 to 113 and the growth of GeneXpert sites from 3 to 31, is fully consistent with WHO recommendations regarding universal rapid diagnostics. The WHO recommendations promote universal access to WHO-approved rapid diagnostics (mWRD) as the initial diagnostic test for all presumptive TB cases. Consequently, the observed rise in bacteriological confirmation and rifampicin resistance detection marks a critical improvement in case finding and reporting, establishing a foundation for achieving the End TB Strategy’s 2030 milestones [14].

A critical objective of the NTRL expansion was to establish in-country capacity for multidrug-resistant and extensively drug-resistant TB testing. Until 2021, limited diagnostic and testing centers offered advanced molecular WHO-approved rapid diagnostics (mWRD), necessitating costly and time-consuming referrals abroad to the national center of the Democratic Republic of Congo. By 2024, 31 DTCs across 11 of 12 departments were equipped to perform mWRD, significantly decentralizing testing and increasing bacteriological confirmation to 73%. With the introduction of a ten-color GeneXpert system and the Xpert MTB/XDR assay, along with culture in late 2024, in-country detection of second-line resistance became possible, facilitating timely treatment initiation and surveillance. The increase in detected cases of MDR and pre-XDR TB reflects improved diagnostic coverage rather than an actual rise in resistance, consistent with observations from other resource-limited settings [15,16]. Together, these innovations have not only curtailed diagnostic delays but also enhanced patient management and treatment follow-up by facilitating more timely and appropriate therapeutic interventions.

To overcome geographic and logistical barriers, the NTRL implemented a national specimen transport system using a hub-and-spoke model, representing one of the most impactful interventions. The network expanded from 123 sputum samples referred from 10 centers in 2022 (first year of implementation) to 5160 samples from 49 centers by 2024. This network expanded geographic coverage and reduced structural gaps in access to molecular testing services.

This network substantially reduced diagnostic inequities between urban and rural populations by ensuring that all presumptive TB patients could access GeneXpert testing, regardless of location. Its successful integration into the national health system demonstrates how logistics platforms for one disease can be leveraged for broader health security, as illustrated by its rapid adaptation to emergency sample transfers for suspected monkeypox cases during the 2024 epidemic. Discussions are ongoing with the national HIV program about potentially integrating viral load and early infant diagnosis specimen transport in the future. The cross-programmatic utilization is consistent with recommendations from the WHO and the Global Fund regarding laboratory network integration, thereby enhancing both efficiency and sustainability [17,18,19].

A systematic integration of HIV testing in TB services represents another key achievement. HIV testing coverage among TB patients increased from 29% in 2016 to 90% in 2024, reflecting WHO guidance on collaborative TB/HIV activities. This integration supports early detection of coinfection, timely antiretroviral therapy initiation, and reduced TB-related mortality [18].

Reliable and accurate diagnostics are essential for patient management and surveillance. By late 2024, the NTRL had established a comprehensive external quality assessment (EQA) framework covering all TB centers nationwide. The Supranational Reference Laboratory in Cotonou (Benin) provides microscopy EQA to the NTRL, Longhorn Vaccines and Diagnostics (USA) oversees GeneXpert EQA to the NTRL, and the NTRL provides both microscopy and GeneXpert EQA to national diagnostic and testing centers. This multi-tiered approach ensures consistent quality and strengthens confidence in national TB surveillance data. While formal performance metrics were not yet available for reporting, this process established the foundation for routine, standardized EQA cycles and continuous laboratory performance monitoring. The two-star WHO SLIPTA rating obtained by the NTRL demonstrates significant progress toward international laboratory accreditation standards [20,21]. Sustaining and improving this rating will require continued mentorship, periodic audits, and investment in quality management systems.

Strategic research partnerships have positioned NTRL as an emerging hub for TB research in Central Africa. Collaborative efforts include national universities, public research institutes, private research foundations, and regional reference laboratories, such as CERMEL (Gabon), Pasteur Institute of Cameroon, and IRESSEF (Senegal). These partnerships enhance research capacity, foster regional knowledge exchange, and facilitate high-quality epidemiological studies.

Nonetheless, several challenges remain. Sustaining infrastructure functionality requires continuous financial investment, reliable reagent and consumable supply chains, and ongoing workforce development to fully operationalize available technologies. The integration of next-generation sequencing (NGS) for molecular surveillance remains underutilized and will be crucial for high-resolution monitoring of transmission dynamics and resistance evolution. Ensuring long-term sustainability, data-driven decision-making, and domestic resource mobilization will be essential to consolidate gains and achieve national TB elimination goals.

Data from India, Uganda, Portugal, and Congo confirms that NTRL investments yield significant public health returns. These investments improve diagnostic access, accuracy, and surveillance quality, even in resource-limited settings. Securing long-term sustainability for the NTRL is both a technical necessity and a strategic imperative for containing TB and strengthening national health security. Continued political commitment, domestic financing, and integration of TB diagnostics into broader disease surveillance frameworks will also be critical to maintaining momentum and achieving national and global End TB targets.

The strengthening of the National Tuberculosis Reference Laboratory in the Republic of Congo illustrates how sustained, system-oriented investments in laboratory infrastructure can support national TB control efforts and offers important policy lessons for other high-burden countries. To consolidate and sustain these gains, national TB strategic plans should formally recognize the NTRL as core public health infrastructure, supported by dedicated and predictable budget lines for operations, maintenance, and essential consumables. These investments must be accompanied by continued development of human resources through structured training, mentorship, and retention strategies, particularly at peripheral levels. In line with WHO and Global Fund recommendations, the diagnostic and quality assurance capacities established for TB also present opportunities for progressive integration into broader health priorities, including antimicrobial resistance surveillance and preparedness for public health emergencies. Finally, strengthened regional collaboration for external quality assessment, technical support, and data-sharing can enhance the efficiency, resilience, and long-term sustainability of national laboratory networks.

This study has several limitations that should be considered when interpreting the findings. First, the analysis relied exclusively on aggregated routinely collected programmatic data, which limited the ability to perform patient-level analyses or to formally assess equity, turnaround times, or clinical outcomes. Second, some relevant indicators, such as specimen transport turnaround times, detailed external quality assessment performance scores, and patient-level outcomes, were not systematically captured during the study period and, therefore, could not be analyzed. Third, the retrospective observational design of this operational evaluation does not allow causal inference; observed improvements over time may reflect a combination of strengthened laboratory organization, increased funding, and broader health system factors rather than the effect of any single intervention. Despite these limitations, the use of standardized national reporting systems and multi-year trend analysis provides a robust overview of programmatic progress and system strengthening at the national level.

## 5. Conclusions

The experience of the National Tuberculosis Reference Laboratory in the Republic of Congo documents substantial changes in laboratory capacity over a seven-year period, including expanded availability of rapid molecular diagnostics and drug susceptibility testing and increased detection of drug-resistant TB. Since 2018, the expansion of national TB reference and coordination structures has occurred alongside greater standardization of diagnostic procedures, broader implementation of external quality assessment, and expansion of specimen referral networks.

From a health policy perspective, these observations highlight the importance of formally resourced national TB institutions with defined responsibilities for laboratory coordination, guideline implementation, workforce training, and data stewardship. Strengthening these institutional functions remains central to maintaining diagnostic capacity, supporting bacteriological confirmation and drug-resistant TB detection, and enabling routine programmatic monitoring in resource-limited settings. The experience presented may inform other high-burden countries seeking to strengthen laboratory networks and program accountability within similar contexts.

## Figures and Tables

**Table 1:** Key performance indicators from 2018 to 2024 (7 annual observations).

Indicator	2018	2019	2020	2021	2022	2023	2024	APC (95%CI)
Treatment success (%)	70	62	71	75	78	81	87	+4.4% (95% CI 1.9 to 7.1; *p* = 0.007)
Lost to follow-up (%)	15.1	21.3	17.3	12.5	11.1	10.2	8.0	−12.7% (95% CI −19 to −5.1; *p* < 0.05).
HIV testing among TB patients (%)	18.2	10.8	42.7	41.9	56.0	82.2	92.0	+26.2% (95% CI 8.8 to 46.5; *p* = 0.007)
Diagnostic and treatment centers with TB testing (n)	38	41	41	44	73	85	113	+10.9% (95% CI 2.5 to 20.0; *p* = 0.016)
GeneXpert machines/sites (n)	3	3	3	3	14	28	31	+60% (95% CI 16 to 137; *p* = 0.01)
Bacteriological confirmation rate (%)	34	37	37	40	43	52	70	+6.8% (95% CI 2.5 to 11.4; *p* = 0.006)

**Table 2:** Molecular TB diagnostic cascade using Xpert assays, Republic of Congo, 2022–2024.

Year	Xpert MTB/RIF or Ultra Tests Performed (n)	TB-Positive Detections (n)	Rifampicin-Resistant TB Detections (n)	RR-TB Cases Tested with Xpert MTB/XDR	Drug-Resistance Profiles Detected by Xpert MTB/XDR (n)
2022	11,609	4185	215	/	/
2023	22,300	5890	250	Yes (102/250)	17 mono-resistant; 49 MDR; 38 pre-XDR
2024	27,318	7179	273	Yes (243/273)	67 mono-resistant; 90 MDR; 88 pre-XDR

**Table 3:** Performance of the national TB specimen referral system, Republic of Congo, 2022–2024.

Year	Samples Referred Through National System (n)	Referral Centers Participating (n)	Departments Covered (n)
2022	123	10	2/12
2023	2154	29	8/12
2024	5160	49	11/12

## Data Availability

The original contributions presented in this study are included in the article. Further inquiries can be directed to the corresponding author.
